# Evolutionary History of the Plant Pathogenic Bacterium *Xanthomonas axonopodis*

**DOI:** 10.1371/journal.pone.0058474

**Published:** 2013-03-07

**Authors:** Nadia Mhedbi-Hajri, Ahmed Hajri, Tristan Boureau, Armelle Darrasse, Karine Durand, Chrystelle Brin, Marion Fischer-Le Saux, Charles Manceau, Stéphane Poussier, Olivier Pruvost, Christophe Lemaire, Marie-Agnès Jacques

**Affiliations:** 1 INRA, UMR1345 Institut de Recherche en Horticulture et Semences, Beaucouzé, France; 2 Université d’Angers, UMR1345 Institut de Recherche en Horticulture et Semences, SFR4207 QUASAV, PRES L’UNAM, Angers, France; 3 AgroCampus-Ouest, UMR1345 Institut de Recherche en Horticulture et Semences, Angers, France; 4 CIRAD, UMR PVBMT, Saint Pierre, Cedex Réunion, France; George Washington University, United States of America

## Abstract

Deciphering mechanisms shaping bacterial diversity should help to build tools to predict the emergence of infectious diseases. Xanthomonads are plant pathogenic bacteria found worldwide. *Xanthomonas axonopodis* is a genetically heterogeneous species clustering, into six groups, strains that are collectively pathogenic on a large number of plants. However, each strain displays a narrow host range. We address the question of the nature of the evolutionary processes – geographical and ecological speciation – that shaped this diversity. We assembled a large collection of *X. axonopodis* strains that were isolated over a long period, over continents, and from various hosts. Based on the sequence analysis of seven housekeeping genes, we found that recombination occurred as frequently as point mutation in the evolutionary history of *X. axonopodis.* However, the impact of recombination was about three times greater than the impact of mutation on the diversity observed in the whole dataset. We then reconstructed the clonal genealogy of the strains using coalescent and genealogy approaches and we studied the diversification of the pathogen using a model of divergence with migration. The suggested scenario involves a first step of generalist diversification that spanned over the last 25 000 years. A second step of ecology-driven specialization occurred during the past two centuries. Eventually, secondary contacts between host-specialized strains probably occurred as a result of agricultural development and intensification, allowing genetic exchanges of virulence-associated genes. These transfers may have favored the emergence of novel pathotypes. Finally, we argue that the largest ecological entity within *X. axonopodis* is the pathovar.

## Introduction

Determining the relative part played by geography and ecological specialization in the reproductive isolation between populations of pathogens is still a great challenge [1], [2]. The extent of isolation between pathogen populations is conditioned by the divergence accumulated in allopatry or by the degree of adaptation to hosts. In complete allopatry, genetic reproductive barriers are less likely to be selected than in sympatry [3], and gene flow remains possible for populations that have diverged for a long time (several million years) [4]. Conversely, specialization on hosts greatly contributes to reproductive isolation and limits gene flow. Host specialization was promoted by plant domestication, which led to a reduction of host genetic variability, and by the development of agriculture, which has uniformized crops and increased host density [5]. More recently, the globalization of agriculture has strongly contributed to breaking natural barriers to dispersal, thereby reducing the geographic isolation and increasing opportunities for gene flow. Subsequent propagation of many pathogens allowed secondary contacts between populations, which have diverged in allopatry [6]. Would such a return to sympatry be sufficient to promote gene flow between these populations of pathogens? The study of the relative importance of gene flow and divergence times between populations occupying different hosts then becomes crucial for understanding evolutionary histories and emergences of pathogens [7], [8].

Unlike for sexual eukaryotes, the classical biological species definition does not apply for prokaryotes, which are asexually reproducing organisms. Bacteria, rather, form ecologically isolated units called ecotypes [9]. The pathovar is an infrasubspecific division that was created to group pathogenic bacteria that display the same symptomatology on the same host range [10]. Would the ecological cohesion of the ecotype concept be found in the pathovar concept? The extent of reproductive isolation among pathovars remains to be clarified, because ecological isolation may be associated with reproductive isolation. Bacteria are mainly clonal but can exchange genes or fragments of their genomes through horizontal gene transfer. Such transfers would be interpreted as recombination events in molecular polymorphism studies. Under the biological species concept for eukaryotes, reproductive isolation can be monitored by a strong decrease in recombination rates between species. In prokaryotes, sequence divergence, as a factor potentially involved in the decrease of recombination efficiency, would play only a little role in the genetic cohesion of bacterial species [11].

Recombination rates are not expected to be homogeneous among loci. Niche-neutral genes such as housekeeping genes are expected to recombine more freely than niche-specifying-genes [12]. For the latter, their transfer into a focus population adapted to a different niche implies a fitness cost resulting in negligible recombination rates. Alternatively, some virulence-associated (VA) genes may act as niche-transcending (NT) genes. NT genes confer a gain of fitness when transferred to a new ecotype (adaptive introgression). The increasing genetic exchanges among strains, due to agricultural globalization, may involve horizontal gene transfer of NT (VA) genes. In the latter case, the emergence of strains with new pathological competences becomes likely.

Bacteria belonging to the genus *Xanthomonas* are collectively responsible for diseases on more than 400 different host plants, among which many are economically important crops [13]. The physiological characteristics of *Xanthomonas* are very homogeneous. However, the diversity within this genus is highlighted by pathogenicity and host ranges. Each strain displays a narrow host range, and strains causing similar symptoms on the same host range are grouped into a pathovar [10]. These plant-associated bacteria are not known to colonize other environments such as soil or water. Symptomatic cultivated hosts are generally the best known hosts; weeds and asymptomatic hosts remain difficult to identify. The species *X. axonopodis* (*sensu* Vauterin and collaborators [14], [15]) groups strains that are collectively pathogenic on highly diverse crops. DNA:DNA hybridizations, Rep-PCR, and AFLP genomic fingerprintings show that *X. axonopodis* does not form a coherent group but comprises six groups named 9.1 to 9.6 [16], [17], [18]. Different MLSA-based studies support this delineation into six groups within *X. axonopodis* indicating a strong but undetermined biological signification for these groupings [16], [17], [19], [20], [21], [22]. Up to now, there has been no obvious ecological rationale such as host range, tissue specificity, or geographical origin to the clustering into six groups. So the nature of the evolutionary processes at the origin of these six groups as well as the extent of isolation between them remains unknown. In addition, within each of these six groups, numerous pathovars are defined only on a phenotypical basis, making each group pathologically heterogeneous.

To derive the parameters of *X. axonopodis* evolutionary history, we used a coalescent-based analysis. The coalescent framework uses DNA sequence data to infer genealogies of a population sample back in Time to the Most Recent Common Ancestor (TMRCA) [23]. We used two complementary methods, one taking recombination and clonal inheritance into account [24], [25] and the other assuming a model of divergence with migration [26] particularly well suited for pathogen diversification [5]. We address the following questions: does the coalescent analysis divide *X. axonopodis* into six groups as other methods did? If so, what is the history of divergence between these groups? What is the most likely mechanism of reproductive isolation for these groups: geography or ecology? Ancient ecological specialization would produce a pattern of divergence matching with host-divergences, whereas geography and/or recent host-jumps may reflect a divergence pattern independent from host divergence. Finally, would the recent increase in agriculture and human-based transfers have favored gene exchanges between formerly divergent populations?

## Materials and Methods

### Strain Sampling

A collection of 131 strains of *X. axonopodis* belonging to 21 pathovars with valid names was selected from the French Collection of Plant Pathogenic Bacteria (CFBP, Angers, France, http://www.angers.inra.fr/cfbp/index_e.html) (Table 1). Most of these pathovars are monophyletic on the basis of previous MLSA-based studies. However, pathovar *dieffenbachiae* is composed of two different clusters of strains displaying different pathological specificities that should be considered as distinct pathovars but have not yet been defined as such [17]. Pathovar *phaseoli* is also genetically diverse and is composed of four genetic lineages, each harboring different sets of genes involved in pathogenicity [27], [28]. Thus 25 genetic lineages that we consider as 25 pathovars are represented in the present analysis. When possible, not less than five strains were sampled in each pathovar. Samples were collected in different geographical areas, on various hosts and over different years. Bacterial cultures were stored in 40% glycerol at −80°C for long term preservation. They were checked for purity and routinely cultivated on YPGA (yeast extract, 7 g liter^−1^; peptone, 7 g liter^−1^; glucose, 7 g liter^−1^; agar, 18 g liter^−1^) for 2–4 days at 28°C.

**Table 1:** List of *X. axonopodis* strains used in the study.

Pathovar (genetic lineage)	Strain code	Host of isolation	Geographic origin	Year of isolation	Group
pv. *alfalfae*	3835	*Medicago sativa*	Australia	1972	9.2
	3836	*M. sativa*	Sudan	n a	
	3837	*M. sativa*	USA	1965	
	7120	*M. sativa*	Japan	1962	
	7121	*M. sativa*	India	n a	
pv. *allii*	6107	*Allium fistulosum*	Japan	1998	9.2
	6358	*A. sativum*	Reunion Island	1994	
	6359	*A. cepa*	USA	1980	
	6362	*A. cepa*	Brazil	1986	
	6364	*A. sativum*	Cuba	1986	
	6367	*A. cepa*	Barbados	n a	
	6369	*A. cepa*	Reunion Island	1996	
	6376	*A. cepa*	Mauritius	1997	
	6383	*A. cepa*	USA	1983	
	6385	*A. cepa*	South Africa	n a	
pv. *anacardii*	2913	*Mangifera indica*	Brazil	n a	9.6
	2914	*M. indica*	Brazil	n a	
	7240	*Anacardium occidentale*	Brazil	2001	
	7241	*A. occidentale*	Brazil	2004	
	7242	*A. occidentale*	Brazil	2004	
	7243	*A. occidentale*	Brazil	2004	
pv. *aurantifolii*	2866	*Citrus aurantiifolia*	Brazil	1982	9.6
	2901	*C. limon*	Argentina	n a	
	3528	*C. limon*	Argentina	1988	
	3529	*C. limon*	Urugway.	1983	
	3541	*C. aurantiifolia*	Mexico	n a	
pv. *axonopodis*	4924	*Axonopus scoparius*	Colombia	1949	9.3
	5141	*A. scoparius*	Colombia	1949	
pv. *begoniae*	1421	*Begonia* sp.	France	n a	9.1
	2524	*Begonia* sp.	New Zealand	1962	
	5676	*B. rugosa*	Antilles	1988	
	5677	*B. pendula*	France	1991	
pv. *betae*	5852	*Beta vulgaris.*	Brazil	1973	9.2
pv. *bilvae*	3136	*Aegle marmelos.*	India	1980	9.5
pv. *citri*	1209	*C. grandis*	Hong-Kong	1963	9.5
	1814	*Citrus* sp.	Reunion Island	1978	
	2525	*C. limon*	New Zealand	1956	
	2900	*Citrus* sp.	Japan	n a	
	3369	*C. aurantifolia*	USA	1989	
	3530	*C. limon*	Urugway.	1984	
	5280	*C. hystrix*	Thailand	1998	
	5284	*Citrus* sp.	Malaysia	1999	
	JK2-20*	*C. aurantifolia*	Saudi Arabia	1988	
	JS582*	*C. aurantifolia*	Iran	1997	
	JJ60-1*	*C. aurantifolia*	India	1988	
	JF90-8*	*C. aurantifolia*	Oman	1988	
	LB302*	*C. aurantifolia* x *C. macrophylla*	USA	2002	
	306 **	n a	n a	n a	
pv. *citrumelo*	3114	*Poncirus trifoliata* x *C. paradisi*	USA	1984	9.2
	3371	n a	n a	1989	
	3841	*P.trifoliata* x *C. sinensis*	USA	n a	
	3842	*P. trifoliata* x *C. paradisi*	USA	n a	
	3843	*C. paradisi*	USA	n a	
pv. *dieffenbachiae* GL A	3132	*Diffenbachia* sp.	USA	1950	9.6
pv. *dieffenbachiae* GL C	3133	*Anthurium* sp.	Brazil	1965	9.4
	5688	*A. andreanum*	Venezuela.	n a	
	5691	*Anthurium* sp.	Mauritius	n a	
pv. *glycines*	1519	*Glycine hispida*	Zimbabwe	1962	9.5
	2526	*G. hispida*	Sudan	1956	
	7119	*G. max*	Brazil	1981	
pv. *malvacearum*	2035	*Gossypium hirsutum*	Argentina	1981	9.5
	2530	*G. hirsutum*	Sudan	1958	
	5700	*G. hirsutum*	Senegal	1990	
	5701	*G. hirsutum*	Madagascar	1990	
	5726	*G. barbadense*	Sudan	1991	
pv. *mangiferaeindicae*	1716	*M. indica*	India	1957	9.5
	2915	*M. indica*	South Africa	1971	
	2933	*M. indica*	Reunion Island	1981	
	2935	*M. indica*	Australia	1978	
	2939	*Schinus terebenthifolius*	Reunion Island	1987	
	2940	*S. terebenthifolius*	Reunion Island	1987	
	7236	*M. indica*	Japan	1993	
	7237	*S. terebenthifolius*	Reunion Island	1994	
	7238	*S.terebenthifolius*	Reunion Island	1994	
	7239	*S. terebenthifolius*	Reunion Island	1994	
pv. *manihotis*	1851	*Manihot esculenta*	USA	n a	9.4
	1860	*M. esculenta*	Nigeria	1978	
	1865	*M. esculenta*	Congo	1977	
	2603	*M. esculenta*	Colombia	1972	
	2624	*M. esculenta*	Reunion Island	1986	
	6544	*M. esculenta*	Brazil	1992	
pv. *phaseoli* GL *fuscans*	1815	*Phaseolus* sp.	Greece	1978	9.6
	1845	*Phaseolus* sp.	Greece	1978	
	4834	*Phaseolus vulgaris*	France	1998	
	6165	*P. vulgaris*	Canada	1957	
	6166	*P. vulgaris*	South Africa	1963	
	6167	*Phaseolus* sp.	USA	1964	
	6960	*P. vulgaris*	Reunion Island	2000	
	6965	*P. vulgaris*	n a	n a	
	6969	*P. vulgaris*	Tanzania	2001	
	6970	*Phaseolus* sp.	USA	1990	
	6971	*Phaseolus* sp.	Tanzania	1992	
	6975	*Phaseolus* sp.	France	1994	
	6976	*Phaseolus* sp.	Czech Republic	1994	
	6979	*P. vulgaris*	Tanzania	2001	
pv. *phaseoli* GL1	412	*P. vulgaris*	USA	n a	9.4
	6164	*P. vulgaris*	Romania	1966	
	6546	*P. vulgaris*	USA	n a	
	6982	*P. vulgaris*	Reunion Island	2000	
	6983	*P. vulgaris*	Reunion Island	2000	
	6984	*P. vulgaris*	Reunion Island	2000	
	6985	*P. vulgaris*	Reunion Island	2000	
pv. *phaseoli* GL2	6988	*P. vulgaris*	Reunion Island	2000	9.6
	6990	*P. vulgaris*	Reunion Island	2000	
	6991	*P. vulgaris*	Reunion Island	2000	
pv. *phaseoli* GL3	6992	*P. vulgaris*	Reunion Island	2000	9.6
	6994	*P. vulgaris*	Tanzania	1990	
	6996	*P. vulgaris*	Reunion Island	2000	
	6993	*P. vulgaris*	Reunion Island	2000	
pv. *ricini*	5863	*Ricinus communis*	Brazil	1981	9.2
	5864	*R. communis*	Brazil	1995	
	5865	*R. communis*	Brazil	1995	
	6541	*R. communis*	Brazil	1981	
	6542	*R. communis*	Brazil	1985	
pv. *spondiae*	2547	*Spondias dulcis*	Mauritius	1985	9.1
pv. *vasculorum*	1289	*Saccharum officinarum*	Reunion Island	1970	9.3
	5696	*Thysanolaena maxima*	Reunion Island	n a	
	5822	*S. officinarum*	Australia	1946	
	5823	*S. officinarum*	Mauritius	1979	
pv. *vesicatoria*	75-3**	*Lycopersicon esculentum*	n a	n a	9.2
	1604	*Capsicum annuum*	Guadeloupe	n a	
	2484	*L. esculentum*	Guadeloupe	1980	
	5594	*L. esculentum*	Guadeloupe	1993	
	5618	*C. annuum*	USA	n a	
	6817	n a	Thailand	1997	
	6864	*C. frutescens*	USA	1947	
pv. *vignicola*	7110	*Vigna unguiculata*	Zimbabwe	n a	9.6
	7111	*V. sinensis*	USA	1942	
	7112	*V. unguiculata*	USA	1942	
	7113	*V. unguiculata*	Sudan	1966	
	7115	*V. sinensis*	Brazil	1978	

CFBP (French Collection of Plant Pathogenic Bacteria) code for strain (Strain code) except * which were provided by O. Pruvost, Cirad, Reunion Island, France and ** code of the reference strains whose genome are publicly available. All strains were provided by the CFBP. Genetic group number (Group) from Rademaker *et al*. (2005); not available (n a).

### DNA Extraction

Suspensions made from fresh cultures (overnight growth at 28°C under agitation in YP broth: yeast extract, 7 g liter^−1^; peptone, 7 g liter^−1^; pH 7.2) were used for DNA extraction using the standard hexadecyltrimenthylammonium bromide method [29]. Quality and quantity of DNA were spectrophotometrically (Nanodrop ND-100, Nanodrop Technologies) adjusted. Then, the extracted DNAs were aliquoted and stored at −20°C in microtubes.

### Gene Amplification and Sequencing

Primers for partial sequencing of seven housekeeping genes (*atpD*: ATP synthase beta chain, *dnaK:* encoding the 70-kDa heat shock protein, *efp*: elongation factor P, *fyuA* coding a transmembrane protein (Ton-B dependent transporter), *glnA:* glutamine synthetase I, *gyrB*: DNA gyrase subunit B, and *rpoD*: RNA polymerase sigma 70 factor) were designed (Table 2) from genomic *Xanthomonas* sequences available in GenBank: *X. axonopodis* pv. *vesicatoria* AM039952 and *X. axonopodis* pv. *citri* AE008923. PCR amplifications were performed in a 50 µl reaction mixture containing 1X Go Taq Buffer (Promega), 200 µM dNTP, 0.5 µM of each primer (Table 2), 0.4 U of Go Taq Polymerase, and 3 ng of template genomic DNA in an Applied Biosystems thermocycler with an initial denaturation at 94°C for 2 min, 30 cycles of denaturation for 1 min at 94°C, annealing for 1.5 min at a gene specific temperature (Table 2), extension for 1.5 min at 72°C, and a final extension for 10 min at 72°C. Purity and yield of PCR products were checked by running 8 µl reaction mixtures in 1.2% agarose gel and post-staining using ethidium bromide. The remaining amplified PCR products were sequenced with reverse and forward primers at the Biogenouest platform (Nantes, France).

**Table 2:** Primers characteristics.

Locus	Sequence of forward (F)and reverse (R) primers (5′ → 3′)	Fragment length (bp)	Annealing temperature (°C)
*atpD*	F: GGGCAAGATCGTTCAGAT	868	60
	R: GCTCTTGGTCGAGGTGAT		
*dnaK*	F: GGTATTGACCTCGGCACCAC	1034	60
	R: ACCTTCGGCATACGGGTCT		
*efp*	F: TCATCACCGAGACCGAATA	445	62
	R: TCCTGGTTGACGAACAGC		
*fyuA*	F: ACCATCGACATGGACTGGACC	963	60
	R: GTCGCCGAACAGGTTCACC		
*glna*	F: ATCAAGGACAACAAGGTCG	1094	60
	R: GCGGTGAAGGTCAGGTAG		
*gyrB*	F: TGCGCGGCAAGATCCTCAAC	1051	60
	R: GCGTTGTCCTCGATGAAGTC		
*rpoD*	F: ATGGCCAACGAACGTCCTGC	1313	60
	R: AACTTGTAACCGCGACGGTATTCG		

### Sequence Acquisition and Alignment

Forward and reverse nucleotide sequences were edited and assembled by using PREGAP4 and GAP4 of the Staden Package [30] and then translated and aligned using CLUSTAL W-based subalignment tool with default parameters [31] available in MEGA 4.0.2 program [32] to obtain high quality sequences. Multiple alignments were manually edited using BIOEDIT program [33]. Amino acid alignments were transposed back to nucleotide sequence level to obtain a codon-based alignment [33]. Sequences were concatenated following the alphabetic order of the genes ending in a sequence of 5466 bp (1–777 for *atpD*, 778–1674 for *dnaK*, 1675–2034 for *efp*, 2035–2898 for *fyuA*, 2899–3882 for *glnA*, 3883–4683 for *gyrB,* and 4684–5466 for *rpoD*).

The Genbank accession numbers for the partial sequences used in this study are: *atpD*: HQ590543 to HQ590673, *dnaK*: HQ590674 to HQ590804, *efp*: HQ590805 to HQ590935, *fyuA*: HQ590936 to HQ591066, *glnA*: HQ591067 to HQ591197, *gyrB*: HQ591198 to HQ591328, and *rpoD*: HQ591329 - HQ591338.

### Statistical Analyses

All summary statistics and genealogical analyses were carried out at the level of *X. axonopodis*; no outgroup is needed with these methods. Occurrence of intragenic and intergenic recombination was analyzed using the four gametes test of Hudson and Kaplan [34] implemented in DnaSP v.5 [35] and seven non-parametric detection programs implemented in RDP version 3.38 [36]: RDP [37], GENECONV [38], MaxChi [39], Chimaera [40], BootScan [41], SiScan [42], and 3Seq [43]. The analysis was performed with default settings for the different detection methods, and the Bonferroni-corrected P-value cut-off was set at 0.05. Recombination events were accepted when detected with at least three detection methods out of seven. The Web-based service GARD (genetic algorithm for recombination detection) was also used to detect and locate recombination breakpoints [44]. All the sequences of the 131 strains of *X. axonopodis* were analyzed under a coalescent framework, in order to take into account all the nucleotidic variation between sequences [24] and to permit the inference of genetic parameters between populations [26], [45]. Haplotype number (Hap), haplotype diversity (*Hd;* [46]), nucleotidic diversities (*θ_π_*; [46], and *θ_w;_* [47]), and neutrality test results (Tajima’s D: [48] and Fu and Li’s D* and F*; [49]) were obtained for each of the seven genes for each population using DnaSP. Bonferroni’s correction for multiple tests [50] was applied.

Clonal genealogy of the 131 strains was estimated using ClonalFrame [24]. Parameter space was explored using a Markov Chain Monte Carlo (MCMC) simulation of 100,000 iterations, of which 30,000 were considered as burn-in. The interval of genealogy sampling was set at 50, which ensured good independence between successive sampling. A 50% consensus tree was produced. Parameters estimated were the mutational rate *θ*, the intragenic recombination rate *R*, the average length of a recombination event δ, the rate ν of new polymorphism introduced by recombination, and the TMRCA. Two additional statistics, *r/m* and *ρ/θ,* represent, respectively, a measure of the impact of the recombination (r) relative to mutation (m) in the diversity of the sample, and a measure of the rate of recombination per site (ρ) relative to the mutation rate (*θ)*. A first analysis was performed on the global dataset. Additional analyses were performed on each population dataset.

Migration rates and divergence time between clusters and their ancestors inferred by ClonalFrame were estimated using the Isolation-with-Migration model of Hey implemented in IMa2 [26]. It was not possible to analyze the dataset at the scale of the 25 genetic lineages because of computation issues [26] and because the DNA sequence dataset was not large enough. In the IMa2 model, an ancestral population of size N_A_ splits at a time *t* into two populations of respective sizes N_1_ and N_2_, which may occasionally exchange a small number of migrants after divergence. Here, migration refers to homologous recombination events affecting housekeeping genes between strains of different clusters. The genealogy given by ClonalFrame makes it possible to set ancestral nodes for estimations of ancestral effective size and divergence time. As our dataset violates the assumption of no recombination within loci, we used the four gametes-test implemented in DnaSP [35] and identified blocks undergoing recombination. These blocks were discarded and the remaining dataset was divided into a set of 94 non-recombinant loci, as suggested in Hey and Nielsen [51]. Priors for *θ* parameter within each population are provided by estimates obtained using ClonalFrame. Parameter space is explored by a heuristic MCMC exploration, and posterior distributions of each parameter are then generated. We used 30,000,000 MCMC iterations with genealogy sampling performed every 100 iterations. The first 1,000,000 iterations were discarded as burn-in. Mixing and convergence were obtained by running 100 Metropolis coupled-chains with 100 chain swapping attempts between iterations. The number of migrants between populations per generation was obtained using *m* estimated by IMa2 and *θ* estimated by ClonalFrame as it assumes clonal inheritance. A generation time of 0.003 year was used.

Heterochronous sampling of strains allows estimating mutation rates by Beast 1.6.1 [52]. This method was applied to those strains for which year of isolation was known. Comparison of clock models using Bayesian Factors in Tracer 1.5 indicated that the strict clock model best fitted our dataset, relative to the lognormal relaxed model (data not shown). Statistical inferences of mutation rates as well as of demography were also performed under the Extended Bayesian Skyline Plot (EBSP) model [53]. Such a model allows for population size changes, a likely situation for pathogens [54], [55], [56]. As population structure may bias estimations of mutation rates, the latter were estimated for each of the six groups independently. Estimated mutation rates were entered as inputs in IMa2 analyses.

Additionally, IMa2 results previously obtained were compared to results of IMa2 pairwise analyses. We took into account the Clonal Frame genealogy to perform pairwise analyses between populations defined at each node: 9.4 *vs.* 9.1; 9.4–9.1 *vs.* 9.2; 9.5 *vs.* 9.6; 9.5–9.6 *vs.* 9.3; 9.1–9.2–9.4 *vs.* 9.3–9.5–9.6. Reducing the number of parameters led to estimates that were not significantly different with the two approaches, thus strengthening our conclusions.

### Population Clustering

We performed a Bayesian clustering analysis using Structure version 2.3 [57] to estimate the number of populations in our dataset. We used the linkage model, which accounts for linkage disequilibrium between nearby sites that occurs in admixed populations [58]. Thirty independent runs were performed for each value of number of populations K = 1 to 30. Each run consisted of 500,000 Markov Chain Monte Carlo (MCMC) iterations of burn-in followed by 1,500,000 MCMC iterations. Optimal values of K were found using the method developed by Evanno *et al.* [59]. The average cluster membership coefficient was calculated by aligning outputs of the 30 runs of each K-clustering with CLUMPP [60], using the “Greedy” algorithm with random input order and 10,000 permutations.

### Inference of Repertoires of Virulence-associated (VA) Genes at Ancestor Nodes

Repertoires of 107 genes coding different VA proteins (Methyl accepting Chemotaxis Proteins –MCPs-, sensory proteins of two component regulatory systems, adhesins and Type III Effectors -T3Es-) were determined, for each strain of this study in previous studies, by Hajri *et al*. [27] and Mhedbi-Hajri *et al*. [28]. Using the Mesquite 2.5 system for phylogenetic computing [61], the matrix of presence/absence of these genes for each strain was associated to the scenario for genealogy obtained in ClonalFrame. Repertoires of genes at nodes of the ClonalFrame genealogy were inferred by parsimony using the Trace Character History function implemented in Mesquite.

We explored the possibility that recombination events, identified by IMA2 on the basis of analysis of seven housekeeping genes, may have occurred with the transfer of some genes belonging to the repertoires of VA genes. Indeed, for each migration event between strains identified by IMA2, we assigned a node considered as donor and a node considered as recipient. We considered that a character may have been horizontally transferred when it is present in the donor, absent in the recipient, but present in descendants of the recipient node. Such an acquisition would correspond to a niche-transcending adaptation [12].

## Results

### A Two-step Evolutionary History within *X. axonopodis*

**Figure 1:**
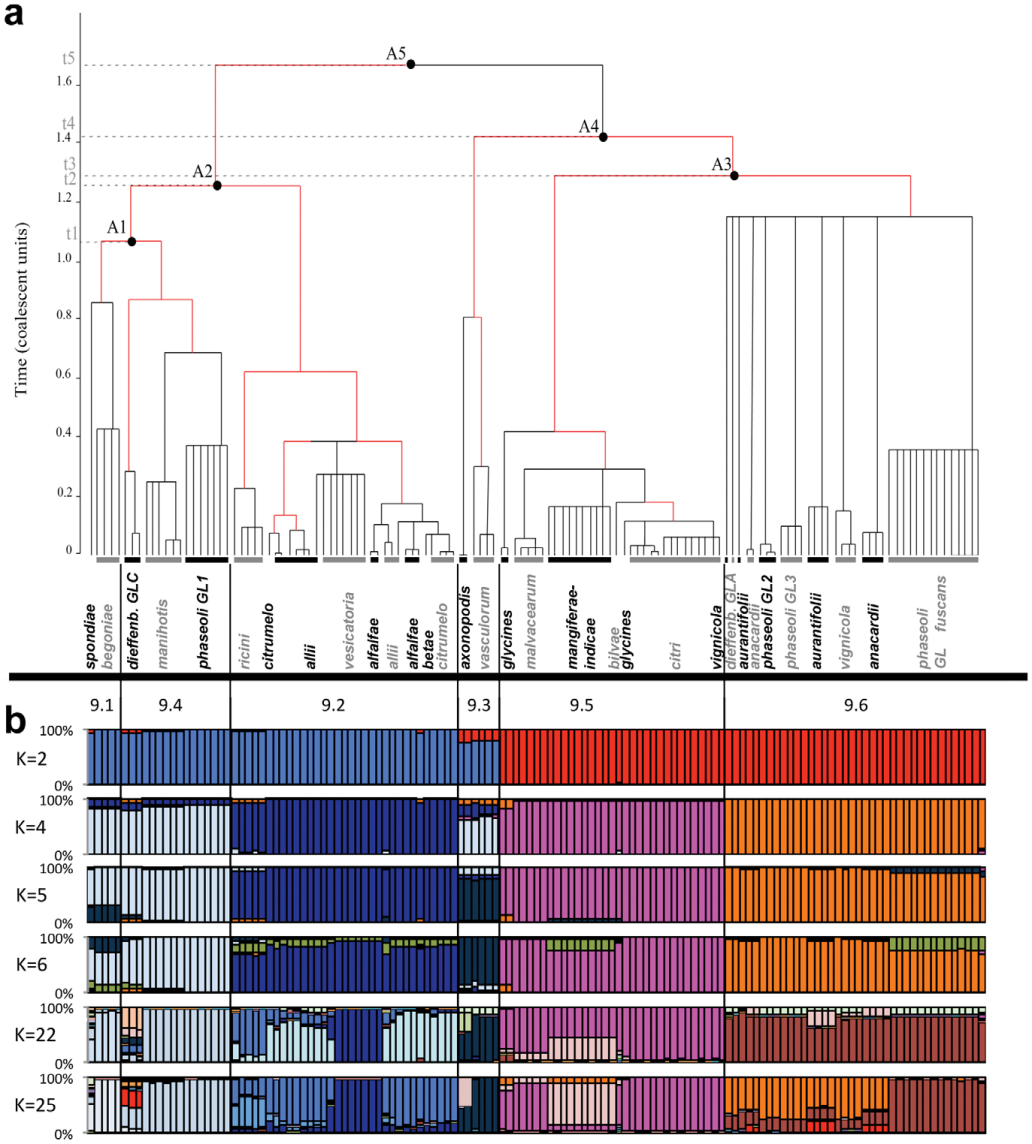
Genetic structure of *X. axonopodis*. (a) Majority-rule consensus genealogy inferred by ClonalFrame. Captions 9.1 to 9.6 refer to genetic groups within *X. axonopodis*. Populations A1 to A5 represent ancestral populations. Parameters were estimated from the sampling of 100 000 iterations of 131 strains typed on 94 non-recombinant loci. Red branches indicate significant occurrence of recombination (p>0.9). Despite being grouped in the same rake, strains may contain different closely related haplotypes. Limit of node age was set at 1.2 in coalescence unit. (b) Cluster analysis using STRUCTURE 2.3 with Linkage model. Four clustering are shown here for K = 2, 4, 5, 6, 21, 22 and. For each K, 30 independent runs were performed and analyzed using CLUMPP with 10,000 permutations. For each run, we used ten independent chains assuming different starting points, and for each chain 5.10^5^ steps for burnin followed by 1.5 × 10^6^ iterations with a thinning interval of 10 steps. Strains appear in the same order as in the ClonalFrame coalescent.

Combining the genealogy and Bayesian clustering approaches revealed an overlapping of two levels of clustering. The clustering of *X. axonopodis* strains into six groups corresponding to rep-PCR groups [17] is strongly supported by the majority-rule genealogy inferred from ClonalFrame analysis (Figure 1). This analysis clustered groups 9.1, 9.2, and 9.4 in one clade and groups 9.3, 9.5, and 9.6 in another clade. In the former clade, group 9.2 diverged first and then 9.1 and 9.4. In the latter clade, group 9.3 diverged first and then 9.5 and 9.6 (Figure 1). However, branching of 9.3 with 9.5 and 9.6 was less robust than other nodes (data not shown). Stricter consensus (*i.e.* with nodes that appeared in more than 60% of cases in the posterior genealogy distribution) genealogies put 9.3 at the same level as the common ancestor; the other nodes remained valid. Alternatively to the genealogy, Structure analysis using K = 2 revealed clustering of 9.3 with 9.1, 9.2 and 9.4 which is disputable according to lower assignment probabilities (Figure 1). Using K = 6 on the overall data set, strains were mainly assigned to five clusters. Indeed, Structure analysis could not differentiate groups 9.1 and 9.4. Combination of both clustering methods demonstrated that grouping of strains into groups 9.1 to 9.6 represents a first episode of divergence. This episode is not yet achieved as 9.1 and 9.4 did not form totally distinct populations (Figure 1).

A second divergence episode can be observed when looking at pathovars. Genealogy showed that divergence among pathovars could be observed only on terminal branches (Figure 1). Of note, all pathovars (as defined in Material and methods) were restricted to one of the six groups and most formed monophyletic clusters of strains. Within a group, some pathovars formed paraphyletic clusters. This is the case for pathovars *allii* (four clusters), *alfalfae* (two clusters), *citrumelo* (two clusters) in group 9.2, for pathovars *glycines* (two clusters) and *anacardii* (two clusters) in group 9.5, and for pathovar *aurantifolii* (two clusters) in group 9.6. For all groups, Time to the Most Recent Common Ancestor (TMRCA) was below 1.2 coalescent units. A star-like genealogy can be observed within group 9.6. This suggests a putative unique event of radiation within 9.6, whereas in the other groups pathovars emerged more gradually (Figure 1).

### Populations of *X. axonopodis* are at Mutation/Drift Equilibrium

All analyzed loci presented a similar level of polymorphism. A total of 62 haplotypes was found in the dataset using concatenated genes (Table S1). Each haplotype belonged to a single pathovar. Within groups and within pathovars, no obvious trend was observed between sample size and level of polymorphism. The nucleotidic θ_π_ and Watterson θ_W_ diversity estimators among all genes were estimated for groups and pathovars. No significant departures from mutation/drift were detected at each level of clustering, *i.e.* the overall dataset and each of the six groups and pathovars (Table S1). Of note, the nucleotidic diversity θ_π_ was null for 11 out of 21 pathovars, suggesting that they could have emerged recently.

### Recombination is an Important Factor in the Evolution of *X. axonopodis*

Evidence of recombination provides valuable information about the intensity of gene flow among strains. To screen for evidence of recombination within *X. axonopodis*, nine methods were employed. Because each method may be sensitive to different kinds of biases, combining different methods allows for the selection of events detected by most methods. Hence, we considered as reliable those events that are detected by most of the methods. Six of the seven detection methods implemented in RDP3 software detected 41 recombinant haplotypes among 62 haplotypes. These recombination events affected strains from pathovars *alfalfae, allii, betae, citrumelo, dieffenbachiae GL C, manihotis, phaseoli GL1,* and *ricini*. The importance of recombination was confirmed using GARD and DnaSp softwares (data not shown).

Using coalescence analysis, we estimated the relative importance of recombination *vs.* point mutation in the emergence of novel haplotypes. Significant recombination was found in the genealogy of the whole dataset (*R* = 12.43[9.12–15.59]) and in the genealogy of the six groups separately (Table 3). Recombination was found to occur as frequently as point mutation in the history of the 131 strains (*ρ/θ* = 0.97 [0.42–1.84]). The mean tract length of the recombinant fragment (δ) was 554 [403–742] bp. However, the impact of recombination was about three times greater than mutation on the diversity observed in the whole dataset (r/m = 3.18 [1.71–5.38]). The rate of new polymorphism introduced by recombination was about 1% for the whole dataset and ranged from 0.003 in group 9.6 to 0.02 in group 9.2 (Table 3). Recombination occurred on some branches (*p*>90%) (Figure 1).

**Table 3:** ClonalFrame parameters for the six groups (9.1 to 9.6) and the whole *X. axonopodis* dataset.

	*X. axonopodis* group						
Parameter	9.1	9.2	9.3	9.4	9.5	9.6	Whole set
n	5	33	6	16	33	38	131
θ	0.009	13.13	0.21	0.24	0.368	7.21	14.10
	[0.001–0.057]	[4.52–25.79]	[0.002–1.282]	[0.006–0.903]	[0.002–1.705]	[1.33–19.98]	[6.67–22.21]
ν	0.0111	0.021	0.009	0.02	0.005	0.003	0.01
	[0.008–0.015]	[0.015–0.028]	[0.006–0.013]	[0.018–0.036]	[0.003–0.007]	[0.001–0.004]	[0.008–0.011]
R	1.868	4.56	0.23	1.09	3.43	2.21	12.43
	[0.009–7.225]	[2.46–7.93]	[0.002–1.743]	[0.57–1.83]	[1.614–6.01]	[0.123–6.55]	[9.12–15.59]
TMRCA	1.053	1.98	2.296	5.72	1.74	0.97	1.67
	[0.253–2.758]	[0.82–4.19]	[0.746–4.335]	[2.83–10.58]	[0.89–3.42]	[0.52–1.8]	[1.23–2.17]

umber of strains (n); number of mutation events (θ); rate of substitution *via* recombination (ν); number of recombination events (R); estimate of time to the most recent common ancestor (TMRCA); [95% confidence interval].

### Isolation-with-Migration Model Reveals a Deep Evolutionary History and Recent Migration Events

Bayesian inference of mutation rates using heterochronous sampling gave similar results for each gene in each of the six groups (Table 4). The estimated mutation rates per gene and per year were on average 2 × 10^−5^. However, mutation rates in group 9.5 were an order of magnitude lower than in the other groups. Extended bayesian skyline plot (EBSP) analyses indicated strong changes in effective sizes for groups 9.2 to 9.6 (Figure S1). For groups 9.2 to 9.5, population bottlenecks may have occurred between *ca.* 1940s and 1960, and they may have occurred in the 2000s for group 9.6. However, the latter result for group 9.6 may be explained by a lack of statistical power, as most of the strains in this group were isolated around year 2000 (Table 1).

**Table 4:** Mutation rate per gene and per year inferred from EBSP analysis in BEAST for each of the six housekeeping genes in each of the six groups within *X. axonopodis*.

	Mutation rate (× 10^−5^) per gene per year in each group					
	9.1	9.2	9.3	9.4	9.5	9.6
*atpD*	4.77	2.02	2.07	2.11	0.14	2.03
*dnaK*	3.15	1.33	1.76	0.90	0.36	3.55
*efp*	5.62	0.80	8.36	1.48	0.52	3.67
*fuyA*	2.54	1.29	2.25	2.65	0.55	2.48
*glna*	2.05	1.23	1.94	0.60	0.22	1.46
*gyrB*	2.36	1.39	2.81	1.41	0.54	3.34
*rpoD*	3.64	1.76	2.05	2.00	0.44	3.51

**Figure 2:**
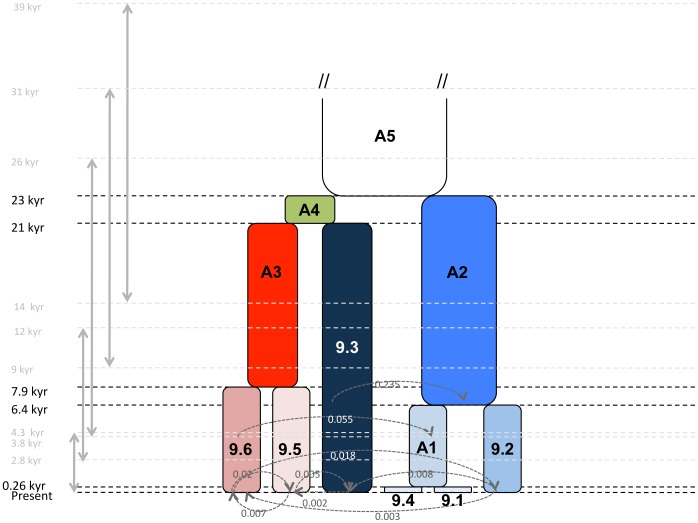
Evolutionary history set in IMa2 of 131 strains of *X. axonopodis* belonging to 25 pathovars. Divergence time estimates are given in kyr at the left (in black). Plain gray double arrows indicate 95% Confidence Interval. Values in light gray r indicate lower and upper limits of 95% highest posterior density on divergence times. Directions of migration are represented by dotted grey arrows; numerical values represent the effective number of migrants. Only migrations significantly different from zero are represented (p<0.05). Populations 9.1 to 9.6 refer to genetic groups in *X. axonopodis* and A1 to A5 refer to ancestral populations.

The evolutionary scenario derived from the genealogical analysis of our strains (Figure 1) was used in IMa2 for parameter estimation (Figure 2; Table S2). Using a generation time of 0.003 year and an average mutation rate per gene per year of 2.10^−5^, divergence times ranged from 0.26 [Present –4.3] thousands of years (kyr) for groups 9.1 and 9.4 to 23.3 [14]–[39] kyr for the most ancestral population. Groups 9.5 and 9.6 diverged 7.9 [3.8–25.8] kyr ago, and group 9.3 diverged from A3 population (common ancestor of 9.5 and 9.6) 21 [9.4–31.5] kyr ago, while groups 9.2 diverged from A1 population (common ancestor of 9.1 and 9.4) 6.4 [2.8–12] kyr ago. Effective size ranged from 8×10^3^ (group 9.1) to 3.7×10^6^ (A2). Nine migration rates were significantly different from zero. Seven of them have occurred since 0.25 kyr (Figure 2) mainly between groups 9.3, 9.5, and 9.6, which belonged to the same super-clade. Within the other super-clade, no genetic exchanges occurred. Population 9.2 exchanged with populations 9.3 and 9.6.

### Co-occurrence of Transfers of VA Genes and of Gene Flow Events in Housekeeping Genes

**Figure 3:**
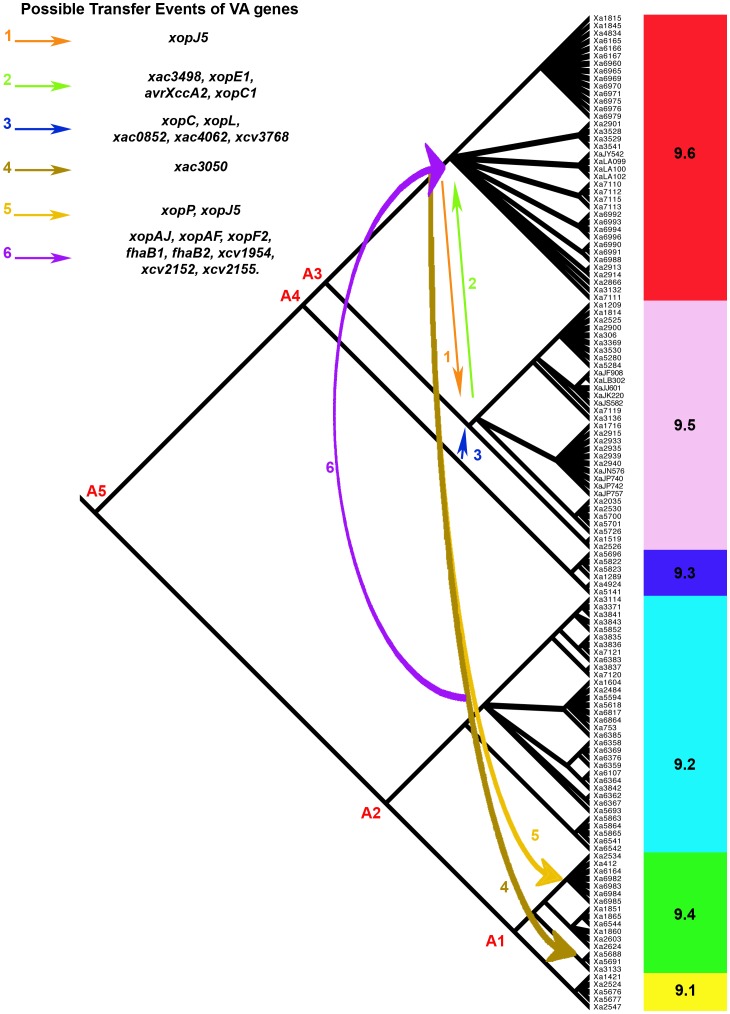
Inferences on transfer of VA genes possibly involved in host specificity. The repertoires of 107 genes encoding MCPs, sensors, adhesins, and T3Es were previously determined for each strain of the collection [27], [28]. Using a parsimony approach implemented in Mesquite 2.5 [61], repertoires of genes at nodes of the ClonalFrame genealogy were inferred. Comparison of repertoires at ancestral nodes provided hypotheses on horizontal transfers concomitant to migration events identified in Figure 2. Populations 9.1 to 9.6 refer to genetic groups in *X. axonopodis* and A1 to A5 refer to ancestral populations. Four of the migration events detected by IMa2 may be associated to transfers of genes encoding MCP (*xac3768, xcv1954*), adhesins (*fhaB1*, *fhaB2*), sensors (*xac0852*, *xac2152, xac2155, xac3050*, *xac3498*, *xac4062*), and T3Es (*avrXccA2*, *xopC1, xopE1, xopF2, xopJ5, xopL, xopP, xopAJ, xopAF*).

The recombination events detected in housekeeping genes show that genetic exchanges were possible between some strains belonging to the different groups. Thus we wanted to assess whether other genetic exchanges may have occurred that involved VA genes. Repertoires of VA genes in ancestor strains were inferred by parsimony analysis at all nodes of the genealogy provided by ClonalFrame analysis. Based on these inferred repertoires of VA genes transfer events detected in our approach involved genes encoding T3Es, sensor proteins, MCPs and adhesins (Figure 3). Transfer of genes encoding T3Es occurred between ancestors of groups 9.5 and 9.6, 9.3 and 9.5, and 9.2 and 9.6. Also, transfer of the T3Es genes *xopP* and *xopJ5* occurred between the ancestors of group 9.6 and of the Genetic Lineage 1 of pathovar *phaseoli*. Transfer events involving genes encoding components of sensory systems occurred between ancestors of groups 9.2 and 9.6, 9.3 and 9.5, and 9.5 and 9.6. Also, gene *xac3050* encoding a sensor protein was transferred from the ancestor of group 9.6 to the ancestor of genetic lineage C of pathovar *dieffenbachiae*. Finally, genes *fhaB1* and *fhaB2* encoding non-fibrillar adhesins were transferred between ancestors of groups 9.2 and 9.6.

## Discussion

Our analyses based on coalescence and Bayesian clustering approaches bring new insight into clusterings within *X. axonopodis.* The scenario suggested by our data involved a first step of diversification of generalist pathogenic bacteria followed by a second step of ecology-driven specialization, *i.e.* host-associated specialization. Secondary contacts between specialized strains probably occurred recently as a result of agricultural intensification and globalization. Subsequently, shuffling of VA genes by genetic exchanges may have favored emergence of novel pathotypes.

Our scenario involves the estimation of divergence times and demographic parameters. Such estimation is based on values of mutation rates that we inferred from our dataset [52]. These values are among the highest ones estimated so far for human bacterial pathogens, as reported by Nübel and colleagues [62]. Calibrating the rate of sequence divergence based on arbitrary values for mutation rates [63] distorts the estimation of divergence times towards millions of years for *Campylobacter coli* [64]. Alternatively, a method based on intra-specific variation leads to estimates that better link genetic structure of populations to the history of ecological niches [23], [64], [65], [66]. Using Beast on our dataset enabled us to estimate ecologically meaningful divergence times between pathovars within *X. axonopodis*, thereby establishing a pioneer framework for scaling evolutionary history of plant pathogenic bacteria, as was recently described for the other bacterial model in plant pathology: *Pseudomonas syringae* [67].

### Episode 1: Diversification of Generalist Pathogenic Bacteria

Nucleotidic variation found in the seven housekeeping genes shows that *X. axonopodis* clustered into five main groups independently from their contemporary hosts and from geography. According to the Isolation-with-Migration model, the five groups coalesced over a period of 25,000 years (Figure 2). The divergence between 9.1 and 9.4, if it exists, can be considered as not fully achieved because the confidence interval of the time of their split contains the present time. The evolutionary parameter inference we provided here allows refining the status of the groups defined earlier on a phylogenetic basis [17], [21].

Geographical isolation does not appear to be the most obvious mechanism of divergence among these five groups. Indeed, no correlation was found between strain sampling localization and assignment to one of the groups. In most groups, strains were isolated in up to four continents. Also, the divergence of groups cannot be explained by the localization of the center of origin and/or domestication of the contemporary hosts of the strains. As an illustration, group 9.6 harbors strains pathogenic on *Citrus* spp. and *Phaseolus* spp. that originate from southeastern Asia and Andean - Mesoamerican centers, respectively [68].

An evolutionary history that is apparently independent from pathological specificities would be a surprising result, as one usually expects evolution of pathogenic bacteria to be driven by host-specificity [69], [70], [71], [72]. Ecological divergence can be used to explain the early divergence of group 9.3; however, host-specificity cannot explain the pattern of divergence between the four other groups. In our collection, group 9.3 contains only strains that are pathogenic on monocots whereas all other groups harbor only strains that are pathogenic on dicots, the only exception being pathovar *allii* in group 9.2. Parkinson and colleagues [19] proposed that *Xanthomonas* genus first arose as a monocot pathogen. A similar proposal was made by Sarkar & Guttman [73] for another plant pathogen: *Pseudomonas syringae.* Group 9.3 seems to have rapidly evolved independently within *X. axonopodis*. For the four other groups in *X. axonopodis*, there is no clear relationship between ecological niches and genealogical clustering. On one hand, bacterial strains that are pathogenic on phylogenetically distant hosts cluster in the same group (Figure 1) [17]. On the other hand, strains that are pathogenic on the same plant species or family are scattered in very divergent groups. For example, pathogens of *Citrus* spp. are found in groups 9.5 and 9.6, which may have diverged around 8,000 yr ago, but also in group 9.2, which diverged from the ancestors of 9.5 and 9.6 around 25,000 yr ago. This is also the case for strains infecting legumes in groups 9.2 (on *Medicago sativa*), 9.4 (on *Phaseolus vulgaris*) and 9.6 (on *P. vulgaris* and *Vigna unguiculata*). In addition, strains that are pathogenic on common bean (*P. vulgaris*) cluster in four lineages [74]. Bean pathogenic strains belonged to groups 9.4 and 9.6, whichcoalesced at a time close to that of the TMRCA (*ca.* 25,000 yr) but that display pathological convergence today.

### Episode 2: an Ecology-driven Specialization

Within each of the five groups, monophyletic clusters are formed by strains displaying different host specialization (Figure 1). Pathovars could then be considered as ecotypes where divergence is mainly driven by ecological isolation [9], which here means isolation by host. Given our data, the most parsimonious hypothesis would be that strains belonging to the various pathovars all derived from a common ancestor carrying a very broad host range. Specialization of strains into pathovars displaying distinct host ranges may have been driven by agricultural development, which arose only recently, beginning about 10 000 years ago, but which intensified recently [68]. Based on our data (Figures 1 and 2), *X. axonopodis* pathovar divergence occurred during the past two centuries. The genetic and environmental uniformity of monocultures coupled with the large size of intensified agro-ecosystems would have selected for highly specialized and aggressive pathogens. The massive increase of agricultural surfaces might have led to large demographic expansion of bacterial pathogens of cultivated plant. Such emergence of the “domesticated” strains through a single founder event has already been reported in other environments [75] Significant increases of population size in the past fifty years have been observed in all *X. axonopodis* groups (Figure S1). No clear signal of such a demographic change has been detected, however, by neutrality tests such as Tajima’s D or Fu and Li’s D* and F* tests. The power of these tests is known to decrease with time after expansion [76]. Expansion of several centuries coupled with a short generation time, as expected in bacteria, would lead to a high number of generations until present and may prevent powerful detection by neutrality tests.

Based on strains collected from symptomatic crops, specialization on host plants appears ecology-driven. However, xanthomonads strains may inhabit and infect wild species [77], [78], or may occur as commensals on plants or may be found in association with seeds [79]. Part of the diversity may therefore have been neglected in our study. However, among non-pathogenic or commensal strains isolated so far from seeds, none belonged to the species *X. axonopodis* (our unpublished data). No xanthomonads have so far been isolated from non-plant environments.

### Episode 3: Secondary Contacts, Occurrence of Genetic Exchanges, and Emergence of Novel Pathotypes?

A surprising result is the occurrence of significant gene flow between some groups in the past three centuries (Figure 2). Agricultural development and globalization may account for secondary contacts allowing recent migration events. Plants traditionally cultivated in different geographical areas and hosting different specialized strains have recently been cultivated in the same areas. Intensive cultivation has probably led to increasing the local effective size of bacterial strains. Then, recombination events with strains from other groups could have been effective. The estimate of *r/m* shows that recombination impacted the diversity of our dataset three times more than mutation (Table 3). Our estimate of r/m = 3.18 for *X. axonopodis* is comparable to that of *Campylobacter insulaenigrae* (*r/m* = 3.2), which is ranked 17^th^ highest among 48 species of bacteria (min. 0.02, *Leptospira interrogans*; max. 63.6, *Flavobacterium psychrophilum*) [80]. This ratio is similar to the one found in a diverse collection of *Xylella fastidiosa* strains [81]. Recombination was found to contribute 5.8 times more than mutation to variation within closely related isolates of *P. syringae* pv. *tomato* [82], contrasting with the results obtained when distantly related isolates were analyzed within the complex *P. syringae* species [73]. In the latter case, mutation was dominant over recombination in generating variation among individuals. Although the genetic distances between isolates in the *P. syringae* strain collection analyzed by Sarkar & Guttman [73] and ours are similar, the impact of recombination in genetic variation is opposite. It is tempting to hypothesize that this could be a consequence of different life history strategies. Indeed, unlike xanthomonads, pseudomonads are known to spend a significant fraction of their life history outside their plant host, *i.e.* in soil or in water [83], where opportunities to exchange genetic material could be low. For *Pseudomonas,* the frequency of genetic exchanges between strains was increased on plants, thereby enhancing the transfer of VA genes [84].

Sympatry resulting in migration events occurs non-randomly among groups. Gene exchanges that occurred in the past 250 years involved groups that contained strains colonizing the same families of host-plants. For example, both groups 9.5 and 9.6 contain strains able to share the same host plant from Rutaceae [22]. Gene exchanges also concern groups in which host range was shown to overlap. Gent *et al*. [85] reported epiphytic asymptomatic survival of pv. *allii* (group 9.2) on legumes and of pv. *phaseoli* (essentially group 9.6) on onions. This niche overlapping potentially creates a secondary contact allowing genetic exchanges between pathologically specialized pathogens. This hypothesis is supported by migration events we observed between groups 9.5 and 9.6 and between 9.2 and 9.6.

Spatial vicinity of crops may constitute another type of secondary contact. Pathovars *vesicatoria* (group 9.2) and *phaseoli* (groups 9.4 and 9.6) colonize hosts (*Lycopersicon esculentum* and *P. vulgaris*, respectively) coming from the same domestication area in Central and South America. These two pathovars share mobile elements not present in other pathovars of the species. Indeed, a common IS element belonging to a family rarely found in Xanthomonadaceae was identified only in the four pathovars of *X. axonopodis* pv. *phaseoli* and in *X. axonopodis* pv. *vesicatoria* [86]. This could indicate that they somehow share ecological niches allowing horizontal gene transfer.

Lastly, our data suggest that VA genes involved in host specificity (MCPs, adhesins, sensors, and T3Es) were transferred between the *X. axonopodis* groups as niche transcending genes. This does not preclude acquisition of VA genes from more distantly related lineages although acquisition of genes in recent times probably occurred mainly among members of Xanthomonadales and not with other Proteobacteria [87]. Transfers of VA genes occurred between groups that were also affected by homologous recombination of housekeeping genes (Figure 3). Since a correlation between pathological specificity and repertoires of VA genes was demonstrated in *X. axonopodis* [27], [28], such transfers may have impacted the host range of strains and promoted the emergence of strains carrying new pathological specificities. On the basis of the analysis of housekeeping genes, we did not observe any genetic exchanges between strains of the three lineages of pathovar *phaseoli* belonging to group 9.6 and the genetic lineage (GL1) falling in group 9.4. However, earlier genetic exchanges between 9.6 and A1 (ancestor of 9.1 and 9.4) may account for the pathological convergence between strains of the different lineages of the pathovar *phaseoli*. Moreover, our approach detected the transfer of the T3E genes *xopP* and *xopJ5* between the ancestor of strains belonging to group 9.6 and the ancestor of the GL1 of pathovar *phaseoli*. Our attempt to associate homologous recombination events with transfer of VA genes does not mean necessarily that these events were linked but that environmental conditions (niche sharing) were met to favor genetic exchanges. However, homologous recombination most probably is not the only exchange mechanism. Indeed, we identified transposases or remnants of insertion sequences in the vicinity of several newly acquired VA genes (data not shown).

In conclusion, while addressing the evolutionary history of *X. axonopodis*, we identified two main steps of diversification. The first one leads to a clustering of generalist pathogens. This clustering does not seem to be based on host specialization or geography. As our collection is composed of pathogenic strains isolated from symptomatic crops, we may have underestimated the overall diversity within *X. axonopodis*. Adding strains from other sources such as weeds and wild relatives in various environments may help improve our understanding of this clustering. The second evolutionary step leads to ecotypes that are specialized pathotypes grouped according to their host range and their symptomatology. These pathovars emerged recently (260 yr to present [4.3 kyr-present]) by specialization likely through agricultural intensification and expansion. Homologous recombinations of housekeeping genes were identified in the recent history between most of the groups. Virulence associated genes have also been exchanged between different groups of strains in the recent past, maybe favoring the emergence of new specializations. The current context of agriculture globalization (cropping and trade) could lead to multiplying the possibilities of niche sharing among pathovars (sympatry of different pathovars) and in association with the recent occurrence of significant gene flow within *X. axonopodis* strains, could favor the emergence of new pathotypes by horizontal transfer of virulence-associated genes.

## Supporting Information

Figure S1 Model of evolution of effective sizes for groups 9.1 to 9.6 within *X. axonopodis* by Extended Bayesian Skyline Plot analyses.
(TIF)

Table S1 Descriptive statistics for polymorphism in *X. axonopodis* inferred from the analysis of seven housekeeping genes from 131 strains estimated on overall data set (a), each of the 6 groups (b), and pathovars (c). Values for pathovars represented by more than one strain are indicated.
(DOC)

Table S2 IMa2 parameter estimates.
(DOC)
