# Supplementary figures and images for: Evolutionary History of the Plant Pathogenic Bacterium Xanthomonas axonopodis
Source: PLoS One. 2013 Mar 7;8(3):e58474. doi: 10.1371/journal.pone.0058474 (PMC3591321; doi:10.1371/journal.pone.0058474)

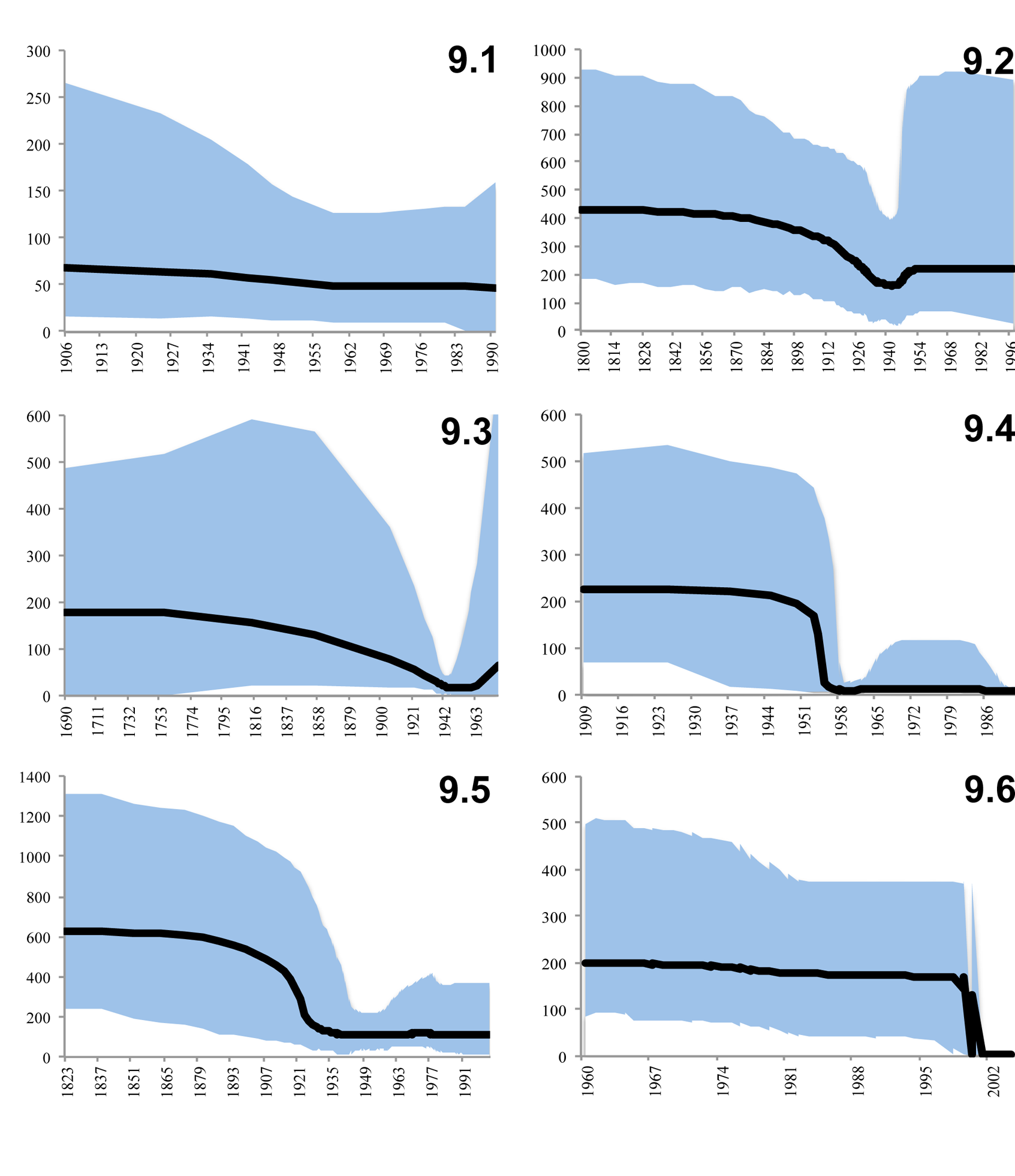

Supplement: Figure S1 — Model of evolution of effective sizes for groups 9.1 to 9.6 within X. axonopodis by Extended Bayesian Skyline Plot analyses. (TIF) [file pone.0058474.s001.tif]
