# Supplementary material for: Evolutionary History of the Plant Pathogenic Bacterium Xanthomonas axonopodis
Source: PLoS One. 2013 Mar 7;8(3):e58474. doi: 10.1371/journal.pone.0058474 (PMC3591321; doi:10.1371/journal.pone.0058474)
Supplement: Table S1 — Descriptive statistics for polymorphism in X. axonopodis inferred from the analysis of seven housekeeping genes from 131 strains estimated on overall data set (a), each of the 6 groups (b), and pathovars (c). Values for pathovars represented by more than one strain are indicated. (DOC) [file pone.0058474.s002.doc]

**Table S1** Descriptive statistics for polymorphism in *X. axonopodis* inferred from the analysis of seven housekeeping genes from 131 strains estimated on: the overall data set (a), each of the 6 groups (b), and pathovars (c).

1. Estimates on the overall dataset

| Locus | Sites | Hap | Hd | S | θ_π_ | θ_W_ | GC% | Tajima’s  D | Fu and Li’s D* | Fu and Li’s  F* |
| --- | --- | --- | --- | --- | --- | --- | --- | --- | --- | --- |
| *atpD* | 777 | 29 | 0.951 | 103 | 0.02226 | 0.02504 | 65.34 | -0.3564 | 1.5092 | 0.8210 |
| *dnaK* | 897 | 32 | 0.948 | 88 | 0.02469 | 0.01944 | 63.15 | 0.8652 | 0.6533 | 0.8958 |
| *efp* | 360 | 18 | 0.867 | 25 | 0.01563 | 0.01491 | 63.17 | 0.1431 | 1.2266 | 0.9525 |
| *fyuA* | 864 | 32 | 0.950 | 111 | 0.02925 | 0.02481 | 64.39 | 0.5769 | 0.6556 | 0.7445 |
| *glna* | 984 | 27 | 0.894 | 85 | 0.01050 | 0.01679 | 63.62 | -1.1970 | -3.9457** | -3.2901 |
| *gyrB* | 801 | 37 | 0.941 | 115 | 0.03014 | 0.02864 | 65.64 | 0.1689 | 1.3641 | 0.9969 |
| *rpoD* | 783 | 34 | 0.959 | 84 | 0.02215 | 0.02196 | 64.34 | 0.0284 | 1.1777 | 0.8083 |
| Total | 5466 | 62 | 0.978 | 611 | 0.02235 | 0.02202 | 64.28 | 0.0493 | 0.4488 | 0.3146 |

Number of analyzed sites (Sites); number of haplotypes (Hap); haplotype diversity (Hd, Nei 1987); number of polymorphic sites (S); nucleotide diversity (θ_π_, Nei 1987); Nucleotide diversity from S (θ_W_, Watterson 1975); GC content (GC%); neutrality tests of Tajima (1989) and Fu and Li (1993) (Tajima’s D and Fu and Li’s D* and F*) and associated P-value: ** *p<0.01*. Fu and Li’s D* on *glna* did not remain significant after application of Bonferroni’s correction for multiple tests with threshold K=0.05/8 = 0.00625.

**(b)Estimates on each of the 6 groups**

| Clade | Locus | n^a^ | Sites^b^ | Hap^c^ | Hd^d^ | S^e^ | θ_π_^f^ | θ_W_^g^ | Tajima’s D^h^ | Fu & Li’s D*^h^ | Fu & Li’s F*^h^ |
| --- | --- | --- | --- | --- | --- | --- | --- | --- | --- | --- | --- |
| 9.1 | *atpD* | 5 | 777 | 2 | 0.400 | 20 | 0.01030 | 0.01236 | -1.2321 | -1.2321* | -1.3200 |
| 9.1 | *dnaK* | 5 | 897 | 2 | 0.400 | 12 | 0.00535 | 0.00642 | -1.2054 | -1.2054 | -1.2776 |
| 9.1 | *efp* | 5 | 360 | 2 | 0.400 | 6 | 0.00667 | 0.00800 | -1.1455 | -1.1455 | -1.1870 |
| 9.1 | *fyuA* | 5 | 864 | 2 | 0.400 | 7 | 0.00326 | 0.00392 | -1.1617 | -1.1617 | -1.2109 |
| 9.1 | *glnA* | 5 | 984 | 2 | 0.400 | 6 | 0.00244 | 0.00293 | -1.1455 | -1.1455 | -1.1870 |
| 9.1 | *gyrB* | 5 | 801 | 2 | 0.400 | 5 | 0.00250 | 0.00300 | -1.1240 | -1.1240 | -1.1558 |
| 9.1 | *rpoD* | 5 | 783 | 3 | 0.700 | 10 | 0.00511 | 0.00613 | -1.1927 | -1.1927 | -1.2578 |
| 9.1 | Concatenated | 5 | 5466 | 3 | 0.700 | 66 | 0.00484 | 0.00580 | -1.2620 | -1.2620** | -1.3693 |
|  |  |  |  |  |  |  |  |  |  |  |  |
| 9.2 | *atpD* | 33 | 777 | 10 | 0.873 | 58 | 0.02168 | 0.01839 | 0.6612 | 0.2215 | 0.4377 |
| 9.2 | *dnaK* | 33 | 897 | 12 | 0.900 | 35 | 0.00813 | 0.00961 | -0.5535 | -1.6268 | -1.5015 |
| 9.2 | *efp* | 33 | 360 | 4 | 0.366 | 12 | 0.00645 | 0.00821 | -0.6867 | -0.9416 | -1.0107 |
| 9.2 | *fyuA* | 33 | 864 | 12 | 0.871 | 51 | 0.01021 | 0.01522 | -1.2110 | -3.1191* | -2.9325* |
| 9.2 | *glnA* | 33 | 984 | 9 | 0.551 | 61 | 0.00530 | 0.01552 | -2.4402** | -4.3451** | -4.3842** |
| 9.2 | *gyrB* | 33 | 801 | 11 | 0.854 | 51 | 0.00777 | 0.01569 | -1.8539* | -3.5897** | -3.5585** |
| 9.2 | *rpoD* | 33 | 783 | 12 | 0.907 | 38 | 0.00718 | 0.01230 | -1.5069 | -2.0230 | -2.1885 |
| 9.2 | Concatenated | 33 | 5466 | 19 | 0.939 | 306 | 0.00958 | 0.01399 | -1.2099 | -2.6447* | -2.5525* |
|  |  |  |  |  |  |  |  |  |  |  |  |
| 9.3 | *atpD* | 6 | 777 | 2 | 0.533 | 7 | 0.00480 | 0.00395 | 1.2674 | 1.5773* | 1.6214 |
| 9.3 | *dnaK* | 6 | 897 | 2 | 0.533 | 6 | 0.00357 | 0.00293 | 1.2465 | 1.5507* | 1.5891 |
| 9.3 | *efp* | 6 | 360 | 3 | 0.733 | 9 | 0.01130 | 0.01217 | -0.4291 | -0.2308 | -0.2949 |
| 9.3 | *fyuA* | 6 | 864 | 2 | 0.533 | 11 | 0.00684 | 0.00561 | 1.3171 | 1.6404** | 1.6996* |
| 9.3 | *glnA* | 6 | 984 | 2 | 0.533 | 9 | 0.00488 | 0.00401 | 1.2971 | 1.6150* | 1.6679 |
| 9.3 | *gyrB* | 6 | 801 | 2 | 0.533 | 16 | 0.01065 | 0.00875 | 1.3468 | 1.6784** | 1.7474* |
| 9.3 | *rpoD* | 6 | 783 | 2 | 0.533 | 6 | 0.00409 | 0.00336 | 1.2465 | 1.5507* | 1.5891 |
| 9.3 | Concatenated | 6 | 5466 | 3 | 0.733 | 64 | 0.00612 | 0.00521 | 1.1147 | 1.4406 | 1.5045 |
|  |  |  |  |  |  |  |  |  |  |  |  |
| 9.4 | *atpD* | 16 | 777 | 4 | 0.750 | 15 | 0.00866 | 0.00582 | 1.9104 | 1.5088** | 1.8666** |
| 9.4 | *dnaK* | 16 | 897 | 4 | 0.692 | 6 | 0.00215 | 0.00202 | 0.2211 | 0.6123 | 0.5809 |
| 9.4 | *efp* | 16 | 360 | 3 | 0.675 | 3 | 0.00368 | 0.00251 | 1.3496 | 1.0443 | 1.2863 |
| 9.4 | *fyuA* | 16 | 864 | 3 | 0.675 | 44 | 0.01914 | 0.01581 | 0.8907 | 1.6674** | 1.6720* |
| 9.4 | *glnA* | 16 | 984 | 3 | 0.675 | 4 | 0.00173 | 0.00123 | 1.2835 | 1.1414 | 1.3501 |
| 9.4 | *gyrB* | 16 | 801 | 4 | 0.692 | 13 | 0.00526 | 0.00489 | 0.2942 | 1.4782* | 1.3241 |
| 9.4 | *rpoD* | 16 | 783 | 3 | 0.675 | 27 | 0.01208 | 0.01049 | 0.6255 | 1.6091** | 1.5375 |
| 9.4 | Concatenated | 16 | 5466 | 5 | 0.767 | 112 | 0.00765 | 0.00625 | 0.9696 | 1.6783** | 1.7071* |
|  |  |  |  |  |  |  |  |  |  |  |  |
| 9.5 | *atpD* | 33 | 777 | 3 | 0.606 | 5 | 0.00256 | 0.00159 | 1.6358 | 1.1348 | 1.4956 |
| 9.5 | *dnaK* | 33 | 897 | 5 | 0.695 | 5 | 0.00218 | 0.00137 | 1.5621 | 0.2416 | 0.7407 |
| 9.5 | *efp* | 33 | 360 | 5 | 0.695 | 4 | 0.00544 | 0.00274 | 2.4714* | 1.0474 | 1.7053** |
| 9.5 | *fyuA* | 33 | 864 | 6 | 0.703 | 23 | 0.00362 | 0.00661 | -1.5689 | -0.3323 | -0.8673 |
| 9.5 | *glnA* | 33 | 984 | 4 | 0.229 | 4 | 0.00047 | 0.00100 | -1.3299 | -1.0136 | -1.2864 |
| 9.5 | *gyrB* | 33 | 801 | 6 | 0.504 | 11 | 0.00244 | 0.00338 | -0.8750 | -0.6208 | -0.8214 |
| 9.5 | *rpoD* | 33 | 783 | 6 | 0.778 | 12 | 0.00504 | 0.00378 | 1.0671 | 0.9890 | 1.1895 |
| 9.5 | Concatenated | 33 | 5466 | 13 | 0.850 | 64 | 0.00282 | 0.00289 | -0.0937 | 0.1469 | 0.0777 |
|  |  |  |  |  |  |  |  |  |  |  |  |
| 9.6 | *atpD* | 38 | 777 | 8 | 0.862 | 14 | 0.00385 | 0.00429 | -0.3229 | 0.1890 | 0.0268 |
| 9.6 | *dnaK* | 38 | 897 | 7 | 0.748 | 9 | 0.00149 | 0.00239 | -1.1077 | -1.1137 | -1.3043 |
| 9.6 | *efp* | 38 | 360 | 3 | 0.240 | 2 | 0.00069 | 0.00133 | -0.9247 | -0.8125 | -0.9782 |
| 9.6 | *fyuA* | 38 | 864 | 8 | 0.799 | 8 | 0.00226 | 0.00222 | 0.0548 | -0.7016 | -0.5447 |
| 9.6 | *glnA* | 38 | 984 | 8 | 0.750 | 13 | 0.00156 | 0.00314 | -1.5890 | -2.2835 | -2.4236 |
| 9.6 | *gyrB* | 38 | 801 | 12 | 0.835 | 16 | 0.00466 | 0.00475 | -0.0632 | -0.0360 | -0.0529 |
| 9.6 | *rpoD* | 38 | 783 | 10 | 0.824 | 12 | 0.00402 | 0.00370 | 0.2683 | 0.4691 | 0.4758 |
| 9.6 | Concatenated | 38 | 5466 | 19 | 0.940 | 74 | 0.00273 | 0.00323 | -0.5647 | -0.7498 | -0.8143 |

Sample size (n); number of analyzed sites (Sites); number of haplotypes (Hap); haplotype diversity (Hd, Nei 1987); number of polymorphic sites (S); nucleotide diversity (θ_π_, Nei 1987); Nucleotide diversity from S (θ_W_, Watterson 1975); neutrality tests of Tajima (1989) and Fu & Li (1993) (Tajima’s D and Fu & Li’s D* and F*) and associated P-value: (* *p<0.05* and ** *p<0.01*). No neutrality test remained significant after a correction of Bonferroni for multiple tests was applied for each clade with threshold K=0.05/8 = 0.00625.

**(c)** Estimates on pathovars. Values for pathovars represented by more than one strain are indicated

| Clade | Pathovar name | N | S | Hap | Hd | θ_π_ | θ_W_ | Tajima’s D | Fu and Li’s D* | Fu and Li’s F* |
| --- | --- | --- | --- | --- | --- | --- | --- | --- | --- | --- |
| 9.1 | *begoniae* | 4 | 1 | 2 | 0.50 | 0.000 | 0.000 | -0.61 | -0.61 | -0.48 |
| 9.2 | *citrumelo* | 5 | 33 | 3 | 0.70 | 0.002 | 0.003 | -1.25 | -1.25 | -1.35 |
|  | *ricini* | 5 | 24 | 3 | 0.70 | 0.002 | 0.002 | -1.24 | -1.24 | -1.33 |
|  | *vesicatoria* | 7 | 0 | 1 | 0.00 | 0.000 | n a | n c | n c | n c |
|  | *alfalfae* | 5 | 76 | 4 | 0.90 | 0.006 | 0.007 | -0.64 | -0.64 | -0.70 |
|  | *allii* | 10 | 64 | 7 | 0.91 | 0.004 | 0.004 | 0.15 | 0.16 | 0.18 |
| 9.3 | *axonopodis* | 2 | 0 | 1 | 0.00 | 0.000 | n a | n c | n c | n c |
|  | *vasculorum* | 4 | 7 | 2 | 0.50 | 0.001 | 0.001 | -0.82 | -0.82 | -0.80 |
| 9.4 | *diffenbachiae* | 3 | 3 | 2 | 0.67 | 0.000 | 0.000 | n c | n c | n c |
|  | *manihotis* | 6 | 1 | 2 | 0.60 | 0.000 | 0.000 | 1.45 | 1.05 | 1.16 |
|  | *phaseoli* GL1 | 7 | 0 | 1 | 0.00 | 0.000 | n a | n c | n c | n c |
| 9.5 | *citri* | 14 | 12 | 5 | 0.66 | 0.001 | 0.001 | 0.68 | 0.38 | 0.53 |
|  | *glycines* | 3 | 24 | 3 | 1.00 | 0.003 | 0.003 | n c | n c | n c |
|  | *malvacearum* | 5 | 6 | 3 | 0.70 | 0.000 | 0.001 | -1.15 | -1.15 | -1.19 |
|  | *mangiferaeindicae* | 10 | 0 | 1 | 0.00 | 0.000 | n a | n c | n c | n c |
| 9.6 | *phaseoli fuscans* | 14 | 9 | 5 | 0.73 | 0.000 | 0.001 | -1.57 | -2.27 | -2.38 |
|  | *phaseoli GL2* | 3 | 2 | 2 | 0.67 | 0.000 | 0.000 | n c | n c | n c |
|  | *phaseoli GL3* | 4 | 0 | 1 | 0.00 | 0.000 | n a | n c | n c | n c |
|  | *vignicola* | 5 | 26 | 3 | 0.70 | 0.002 | 0.002 | -0.17 | -0.17 | -0.18 |
|  | *anacardii* | 6 | 7 | 3 | 0.60 | 0.001 | 0.001 | 0.89 | 1.15 | 1.17 |
|  | *aurantifolii* | 5 | 22 | 4 | 0.90 | 0.002 | 0.002 | -1.24 | -1.24 | -1.33 |

Genetic subgroups 9.1 to 9.6 (Clade); number of strains (N); number of polymorphic sites (S); number of haplotypes (Hap); haplotype diversity (Hd, Nei 1987);nucleotide diversity (θ_π_, Nei 1987); Nucleotide diversity from S (θ_W_, Watterson 1975); neutrality tests of Tajima (1989) and Fu and Li (1993) (Tajima’s D and Fu and Li’s D* and F*); non available (n a); not computable (n c).
