# Supplementary material for: Evolutionary History of the Plant Pathogenic Bacterium Xanthomonas axonopodis
Source: PLoS One. 2013 Mar 7;8(3):e58474. doi: 10.1371/journal.pone.0058474 (PMC3591321; doi:10.1371/journal.pone.0058474)
Supplement: Table S2 — IMa2 parameter estimates. (DOC) [file pone.0058474.s003.doc]

**Table S2** IMa2 parameter estimates

| Parameters ^a^ | HiPt^b^ | HPD95Lo^c^ | HPD95Hi^c^ |
| --- | --- | --- | --- |
| t0 | 256.2 | 0 | 4355 |
| t1 | 6405 | 2818 | 12041 |
| t2 | 7942 | 3843 | 25876 |
| t3 | 21265 | 9479 | 31513 |
| t4 | 23314 | 14091 | 39711 |
| Ne_9.1_ | 8540 | 0 | 145180 |
| Ne_9.2_ | 555099 | 315979 | 777139 |
| Ne_9.3_ | 298899 | 179340 | 572179 |
| Ne_9.4_ | 25620 | 0 | 162260 |
| Ne_9.5_ | 247660 | 128100 | 452619 |
| Ne_9.6_ | 315919 | 179340 | 538019 |
| Ne_A1_ | 1169978 | 606339 | 2673015 |
| Ne_A2_ | 2741335 | 1340777 | 5439970 |
| Ne_A3_ | 42700 | 0 | 1276276 |
| Ne_A4_ | 59780 | 25620 | 15363431 |
| Ne_A5_ | 1460337 | 162260 | 2775495 |
| Nm9.1>9.2 | 0.0001375 | 0 | 0.0021125 |
| Nm9.1>9.3 | 0.0000115 | 0 | 0.0019435 |
| Nm9.1>9.4 | 0.0000125 | 0 | 0.0020875 |
| Nm9.1>9.5 | 0.0000115 | 0 | 0.0019205 |
| Nm9.1>9.6 | 0.0000105 | 0 | 0.0019425 |
| Nm9.2>9.1 | 0.0032775 | 0 | 0.05915 |
| Nm9.2>9.3 | 0.0018105 | 0 | 0.011295 |
| Nm9.2>9.4 | 0.0012285 | 0 | 0.0608 |
| Nm9.2>9.5 | 0.0000265 | 0 | 0.012325 |
| **Nm9.2>9.6** | **0.018795** | **0.00894** | **0.03201** |
| Nm9.2>A1 | 0.00529 | 0 | 0.02398 |
| Nm9.3>9.1 | 0.0000515 | 0 | 0.042485 |
| **Nm9.3>9.2** | **0.008865** | **0.0010695** | **0.022875** |
| Nm9.3>9.4 | 0.0000515 | 0 | 0.041765 |
| **Nm9.3>9.5** | **0.00546** | **0.0000915** | **0.01656** |
| Nm9.3>9.6 | 0.0000265 | 0 | 0.01492 |
| Nm9.3>A1 | 0.0000335 | 0 | 0.022745 |
| Nm9.3>A2 | 0.0000455 | 0 | 0.041635 |
| Nm9.3>A3 | 0.02525 | 0 | 0.045245 |
| Nm9.4>9.1 | 0.0000105 | 0 | 0.0033495 |
| Nm9.4>9.2 | 0.0000095 | 0 | 0.0032395 |
| Nm9.4>9.3 | 0.0000105 | 0 | 0.0032235 |
| Nm9.4>9.5 | 0.0000095 | 0 | 0.0033345 |
| Nm9.4>9.6 | 0.0006195 | 0 | 0.0040215 |
| Nm9.5>9.1 | 0.0000295 | 0 | 0.02646 |
| Nm9.5>9.2 | 0.0016465 | 0 | 0.00727 |
| **Nm9.5>9.3** | **0.0019575** | **0.0000435** | **0.00712** |
| Nm9.5>9.4 | 0.0000295 | 0 | 0.02304 |
| **Nm9.5>9.6** | **0.021175** | **0.007975** | **0.03241** |
| Nm9.5>A1 | 0.0000185 | 0 | 0.008195 |
| Nm9.5>A2 | 0.01531 | 0 | 0.03077 |
| Nm9.6>9.1 | 0.0000395 | 0 | 0.026665 |
| **Nm9.6>9.2** | **0.0031265** | **0.0003515** | **0.009085** |
| Nm9.6>9.3 | 0.0000135 | 0 | 0.0038205 |
| Nm9.6>9.4 | 0.0000395 | 0 | 0.02919 |
| **Nm9.6>9.5** | **0.007115** | **0.0012495** | **0.02272** |
| Nm9.6>A1 | 0.0000825 | 0 | 0.00619 |
| Nm9.6>A2 | 0.0008625 | 0 | 0.03491 |
| NmA1>9.2 | 0.0002405 | 0 | 0.0623 |
| NmA1>9.3 | 0.010685 | 0 | 0.08735 |
| NmA1>9.5 | 0.0014625 | 0 | 0.04924 |
| **NmA1>9.6** | **0.05475** | **0.019885** | **0.1284** |
| **NmA2>9.3** | **0.2349** | **0.07695** | **0.5165** |
| NmA2>9.5 | 0.07895 | 0 | 0.3828 |
| NmA2>A1 | 0.0009995 | 0 | 0.3528 |
| NmA2>A3 | 0.026985 | 0 | 0.3728 |
| NmA2>A4 | 0.1669 | 0 | 0.4288 |
| NmA3>9.3 | 0.0009995 | 0 | 0.6945 |
| NmA3>A2 | 0.0009995 | 0 | 0.6705 |
| NmA4>A2 | 0.0029985 | 0 | 1.1565 |

i^th^ divergence time in years according to the scenario provided in Figure 1 (t_i_); effective size of the population calculated with a generation time equal to 0.003 year (Ne*_i_*); number of migrants from population j to i (*Nmi>j*). Estimates of *Nmi>j* significantly different from zero are indicated in bold. Point with the highest value in the histogram of posterior distribution (*Hipt*); lower (HPD95Low) and higher (*HPD95Hii*) bounds of the estimated 95% highest posterior density.
